# Health Tract No. 339

**Published:** 1868-10

**Authors:** 


HEALTH TRACT No. 339.

### THE LIVER.

Weighing about four pounds, is the great wheel of life's machine; it regulates the whole mechanism of man; when it "acts," works well, every other wheel, gland, factory, works with it; when it stops, is "torpid," the whole system begins to get out of order; the feet become cold; the head aches; the mouth tastes bad; there are pains at the edges of the ribs; at the shoulder blades; on the tops of the shoulders; the body is chilly; the mind is confused; the spirits despondent; the disposition fretful, peevish and complaining; there is no ambition, no animation, no life; and if these things are allowed to go on, especially if moodiness is cherished and melancholy feelings are indulged in, the end is suicide. This unhappy state of mind and body is the result of what is called "biliousness," that is, the liver, whose office it is to withdraw the bile from the blood, fails to perform that duty, and the blood, having more and more bile in it, becomes more and more impure; thickens more and more, until at length it is almost too thick to flow at all; if this take place in the chest, it is called "congestion of the lungs;" if in the liver, "congestion of the liver;" if in the skull, "congestion of the brain;" if in the whole body, it is "congestive fever," which generally means death; some of these congestions, may arise from other states than a disordered liver.

The bile is composed mainly of those waste portions of the human machine, which, having subserved their purpose, are not further needed, but require to be removed from the body; and in the wonderful wisdom and economy of the great Architect of our frame, and of all worlds, the very passing out of this waste, is made to answer a purpose fundamentally essential to all human health; for, after having eaten a meal, the bile is conveyed into the intestinal canal, drop by drop, causing an action which results in the regular daily motion of the bowels, without which there can never be good health for forty-eight hours at a time; hence, "constipation" shows that the liver is not working healthfully, and remedies are required which "act upon the liver." There are two safe, unmedicinal modes of acting on the liver, of starting the machinery of life, when it tends to stand still; go to bed, wrap up warm, make hot applications to the feet, drink warm teas abundantly, so as to cause profuse perspiration for two or three hours; a better plan is, go to work in the open air and keep at it, to the extent of exciting a gentle perspiration until tired or very hungry, for whatever starts perspiration on the skin, starts the wheel of the liver to working, and the person gets well apace.

